# Continued Intravenous Versus First Week Transition to Oral Antibiotic Therapy in Bloodstream Infections: A Systematic Review and Meta-Analysis

**DOI:** 10.7759/cureus.65298

**Published:** 2024-07-24

**Authors:** Cara Mohammed, Hoi Kei Choi, Sana Altaf, Joshua Sajja, Lynda A Ezike, Jada Wang, Urenna O Ihezue, Juan J Prieto, Syeda Simrah Fatima, Adetola G Mowo-wale

**Affiliations:** 1 Orthopaedic Surgery, Sangre Grande Hospital, Sangre Grande, TTO; 2 Neuroscience, University of Michigan, Ann Arbor, USA; 3 Internal Medicine, Deccan College of Medical Sciences, Hyderabad, IND; 4 General Medicine, Siddhartha Medical College, Vijayawada, IND; 5 Internal Medicine, Kursk State Medical University, Kursk, RUS; 6 Internal Medicine, St. George's University, Brooklyn, USA; 7 Public Health, College of Medicine, Imo State University, Owerri, NGA; 8 General Medicine, Universidad Autónoma de Guadalajara, Zapopan, MEX; 9 Internal Medicine, Rajiv Gandhi University of Health Sciences, Bangalore, IND; 10 Internal Medicine, College of Health Sciences, Obafemi Awolowo University, Sagamu, NGA

**Keywords:** meta-analysis, healthcare economics, mortality, treatment success, step-down therapy, oral antibiotics, intravenous antibiotics, bloodstream infections

## Abstract

Bloodstream infections (BSIs) are a major public health concern worldwide, requiring prompt and effective antibiotic therapy. Traditionally, intravenous (IV) antibiotics have been preferred for their rapid action and consistent absorption. However, interest is growing in transitioning to oral (PO) antibiotics when suitable, due to similar pharmacokinetics, improved patient outcomes, and reduced healthcare costs. This meta-analysis aims to evaluate the clinical effectiveness of switching from IV to PO antibiotics for both gram-negative and gram-positive BSIs. Scopus, Embase, and PubMed databases were comprehensively searched until March 2023. The review included randomized controlled trials and cohort studies comparing continued IV therapy with early transition from IV to PO antibiotics within the first week of admission. Inclusion criteria encompassed studies involving adult patients (≥18 years) and reporting specific outcomes such as treatment success, mortality, and hospital readmissions. Meta-analysis of 17 studies comprising 11,245 patients demonstrated higher treatment success rates overall (OR: 1.40, P=0.04), particularly in gram-negative infections (OR: 1.42, P=0.05). However, this effect was not statistically significant in the gram-positive subgroup (OR: 1.41, P=0.036). Oral switch significantly reduced all-cause mortality overall (OR: 0.35, P=0.003), especially in gram-negative infections (OR: 0.22, P=0.008), but not significantly in gram-positive infections (OR: 0.60, P=0.09). Both gram-negative and gram-positive infections benefited from shorter hospital stays (P<0.0001), despite significant heterogeneity. Hospital readmission rates did not significantly differ between IV and oral switch groups (P=0.53). Our meta-analysis suggests potential benefits of early transition from IV to PO antibiotics for BSIs, including improved treatment outcomes and shorter hospital stays without an increased risk of readmission. However, these findings are subject to selection bias, and further standardized randomized trials are essential to validate these results.

## Introduction and background

Bloodstream infections (BSIs) pose a significant public health challenge worldwide, with increased morbidity and mortality [1]. The conventional approach to managing severe infections has relied on intravenous (IV) administration of antibiotics, known for their rapid onset of action and reliable bioavailability. However, there is a growing need to reevaluate and shift towards earlier step-down therapy with oral (PO) antibiotics when clinically appropriate [2,3].

This transition is supported by scientific evidence demonstrating the pharmacokinetic and pharmacodynamic comparability between certain IV and PO antibiotic formulations. For instance, fluconazole and metronidazole exhibit up to 90% bioavailability when administered orally [3]. Moreover, considerations such as patient safety and healthcare economics justify the shift. Oral therapy offers advantages such as reduced risk of catheter-related complications, decreased incidence of healthcare-associated infections, and improved patient mobility [4]. Economically, transitioning to PO antibiotics can lead to substantial cost reductions by reducing hospitalization durations and the need for IV access devices [2].

This meta-analysis was conducted by authors from various countries, facilitated by collaboration within an online medical research community. Currently, there are no systematic reviews and meta-analyses available that summarize the outcomes of continued IV therapy versus early transition to oral therapy. The objective of this meta-analysis is to comprehensively assess the scientific basis, clinical nuances, and broader implications of transitioning from IV to PO antibiotics for both gram-negative and gram-positive systemic infections. 

## Review

Materials and methods

Literature Search

Searches were performed electronically on applications such as Scopus, Embase, and PubMed from the database inception to March 2023. The Population, Intervention, Comparison, and Outcome (PICO) strategy was used to identify studies that compared patients who received continued IV treatment and those who received IV to oral treatment. The updated 2020 version of the Preferred Reporting Items for Systematic Reviews and Meta-Analyses (PRISMA) guidelines was used to ensure a comprehensive search strategy [5].

The keywords used to identify the studies were as follows: (Gram-negative bloodstream infection OR gram-negative sepsis) AND (step-down therapy OR antibiotic therapy) AND (randomized controlled trial OR cohort study OR case-control study), (Bacteremia OR sepsis OR septicemia OR bloodstream infection) AND (intravenous OR IV) AND (Oral) AND (antibiotic).

The studies were screened using Rayyan. The results were initially screened through the titles and abstracts by two groups of three independent authors (SA, AM, and SS and JS, JP, and EA). All conflicts were meticulously reviewed and resolved by another author (CM). Duplicates were also omitted from the total number of titles and abstracts of the accumulated. The final selection of studies was determined using the pre-established inclusion and exclusion criteria.

Inclusion and Exclusion Criteria

Randomized controlled trials, cohort studies, and case-control studies were the studies that were taken into consideration. The inclusion criteria were (1) studies evaluating step-down therapy for gram-negative BSI, (2) studies reporting one of the specific outcomes, (3) patients >18 years old, (4) studies published in English, and (5) antibiotic switch taking place in the first week of admission. Exclusion criteria were (1) non-human studies, (2) studies with no outcome data, (3) case reports, case series, and review articles, and (4) oral switch after the first week of treatment or an undetermined date. The main inclusion criteria were studies that compared outcomes between continued IV and IV to oral group approach. Studies that did not include at least one comparative outcome of interest were excluded from the analysis.

The studies that reported the following outcomes were accounted for (1) clinical resolution, (2) mortality, (3) hospital readmission, and (4) 30-day readmission.

We reviewed a total of 337 studies from PubMed, 703 studies from Embase, and 388 studies from Scopus. Of the 1428, 306 duplicates were removed, and 1022 studies were screened for the criteria abovementioned using Rayyan. Ultimately, 17 studies were included in our systematic review and meta-analysis.

Data Extraction and Outcomes

The data extraction was conducted by five investigators (AM, SA, UI, JS, and EA). The obtained data was revised, and conflicts during the process were resolved by two independent reviewers (HC and AM) ensuring accuracy and consistency. For categorical data, the event and total numbers were extracted for each group, whereas continuous data were coded as means and SDs. If continuous data were reported using median and range/interquartile range, we used the validated formulas by Wan and colleagues to perform appropriate conversions [6]. The extracted data focused on the key demographic characteristics, including age, gender, comorbidities like immunocompromised state, diabetes, presence of a prosthetic device, and source of infection. The main outcomes included short-term (in-hospital or 30-day), all-cause mortality, in-hospital length of stay, treatment failure, and treatment success. 

Quality Assessment

To assess the quality of the included studies, we used the Newcastle-Ottawa scale risk of bias assessment tool. Two independent reviewers (SS and JP) performed the quality assessment by reviewing the articles and checking for the selection criteria, comparability, and outcomes. At the end of this, a final table was constructed based on their agreement. The assessment cut-off for follow-up length was set at 30 days. We regarded follow-up as sufficient if no more than 30 days. Follow-up was considered sufficient if no more than 10% of the patient cohort data were lost.

Statistical Analysis

This meta-analysis adhered to the recommendations provided by the Cochrane Collaboration and Meta-analysis of Observational Studies in Epidemiology (MOOSE) [7]. For data analysis, we used the Review Manager software version 5.4.1 developed by the Cochrane Foundation. The random-effects model and the method of Mantel-Haenszel were utilized to calculate the odds ratio (OR) and the corresponding 95% confidence intervals (CIs) for categorical outcomes. A random model with the inverse variance method was employed to estimate the weighted mean difference in continuous data. A two-sided p-value of less than 0.05 was considered a statistically significant outcome.

Table 1 shows the risk of bias assessment using the Newcastle-Ottawa scale.

**Table 1:** Risk of bias assessment using the Newcastle-Ottawa scale (follow-up length was determined to be 30 days, adequacy of follow-up for 30 days) Stars (★) indicate that the respective study has satisfied this quality measure. Scores <4 indicate poor quality. Scores 4-6 indicate moderate quality. Scores >6 indicate good quality.

Study ID	Selection				Comparability		Outcome			Score
	Representativeness of the exposed	Selection of the non-exposed	Ascertainment of exposure	Outcome of interest not present at the start of the study	Main factor	Additional factor	Assessment	Follow-up length	Adequacy of follow-up	
Broermann et al. [8]	★	★	★	★	★	★	★	★	★	9
Ramos-Otero et al. [9]	★	★	★	★	★	0	★	★	★	8
Nguyen et al. [10]	★	★	★	★	★	0	★	★	★	8
Tamma et al. [11]	★	★	★	★	★	0	★	★	★	8
Tossey et al. [12]	★	★	★	★	0	0	★	★	★	7
Engers et al. [13]	★	★	★	★	★	0	★	0	0	6
Rieger et al. [14]	★	★	★	★	★	0	★	★	★	8
Salam et al. [15]	★	★	★	★	★	0	★	★	★	8
Yetmar et al. [16]	★	★	★	★	★	★	★	★	★	8
Tingsgard et al. [17]	★	★	★	★	★	0	★	★	★	8
Waked et al. [18]	★	★	★	★	★	0	★	★	★	8
Kang et al. [19]	★	★	★	★	★	0	★	★	★	8
Pradubkham et al. [20]	★	★	★	★	★	0	★	★	★	8
Thurber et al. [21]	★	★	★	★	★	0	★	0	0	6
Omrani et al. [22]	★	★	★	★	★	0	★	★	★	8
Gandhi et al. [23]	★	★	★	★	★	0	★	★	★	8
Gillins et al. [24]	★	★	★	★	★	0	★	★	★	8

Results

Included Studies

**Figure 1:**
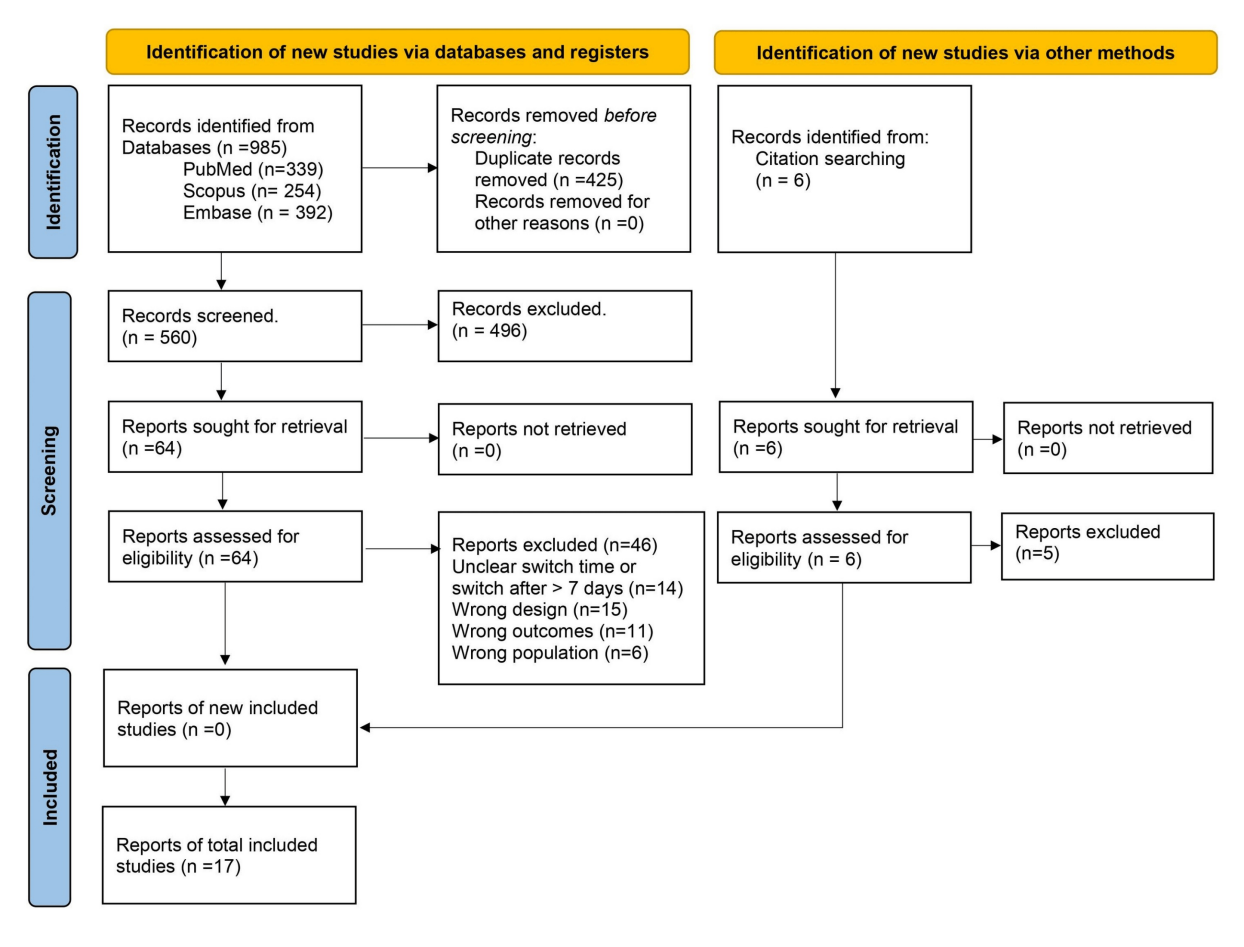
PRISMA flowchart illustrating the different phases in study selection and reasons for exclusion PRISMA: Preferred Reporting Items for Systematic Reviews and Meta-Analyses

Seventeen studies involving 11245 patients (5143 patients switched from IV to PO, while 6102 patients received continued IV) were included in this meta-analysis for the gram-positive and gram-negative studies. Figure 1 shows the PRISMA flowchart illustrating the different phases in study selection and reasons for exclusion. Summary of included studies characteristics is shown in Table 2.

**Table 2:** Summary of study characteristics IV: intravenous; PO: oral; BSIs: bloodstream infections; HIV: human immunodeficiency virus; SSTIs: skin and soft tissue infections

Study ID	Study period	Organism (gram-positive versus gram-negative)	Study design	Number of patients		Study main conclusion
				IV	IV-PO	
Broermann et al. 2022 [8]	2016-2020	Gram-positive	Retrospective cohort	99	123	There is no difference in the risk of treatment failure in patients with uncomplicated streptococcal BSIs treated with partial PO antibiotic therapy compared to standard IV therapy. Partial PO antibiotic therapy may reduce the length of hospital stay.
Ramos-Otero et al. 2022 [9]	2017-2019	Gram-positive	Retrospective cohort	47	51	IV-to-PO step-down therapy for uncomplicated streptococcal BSIs was both safe and effective compared to treatment with IV-only therapy. Additionally, IV to PO was associated with significantly shorter lengths of hospital stay.
Nguyen et al. 2023 [10]	2019-2020	Gram-negative	Retrospective cohort	114	85	PO step-down therapy was not associated with increased 30-day all-cause mortality. It was also more cost-effective than IV-only therapy, while both groups had similar bacteremia recurrence within 30 days.
Tamma et al. 2019 [11]	2008-2014	Gram-negative	Retrospective cohort	1285	876	Until a clinical trial is performed, study findings suggest that PO step-down therapy is not associated with inferior clinical outcomes for patients with *Enterobacteriaceae* bacteremia who have received appropriate source control and demonstrated an appropriate clinical response.
Tossey et al. 2021 [12]	2011-2017	Gram-negative	Retrospective cohort	133	78	The use of PO switch may be considered for the definitive treatment of uncomplicated *Enterobacterales* BSI in cancer patients.
Engers et al. 2024 [13]	Jan 2019-Dec 2019	Gram-negative	Retrospective cohort	2612	1969	Success rates with earlier PO antibiotic are superior when compared to continued IV because most patients demonstrated clinical stability by day 5.
Rieger et al. 2017 [14]	2010-2015	Gram-negative	Retrospective cohort	106	135	IV transitioned to PO treatment was associated with a shorter length of stay and fewer hospital antibiotic days compared with IV-only therapy in patients with bacteremic *Enterobacteriaceae* urinary tract infection.
Salam et al. 2022 [15]	N/A	Gram-positive	Retrospective cohort	36	43	PO step-down therapy may be non-inferior to IV therapy for clinical success with a trend towards decreased length of hospital stay and line complications. However, this study was not powered to meet statistical significance.
Yetmar et al. 2023 [16]	2015-2020	Gram-positive	Retrospective cohort	66	66	PO switch is not non-inferior to continued IV treatment in patients with beta-hemolytic streptococcal BSI; rather, there is a worse 30-day outcome. This difference in outcomes may be related to the choice of PO beta-lactam agents with limited bioavailability.
Tingsgard et al. 2024 [17]	2018-2021	Gram-negative	Retrospective cohort	481	433	The mortality associated with early antibiotic step-down treatment is comparable to that associated with receiving prolonged IV antibiotic treatment for individuals with uncomplicated gram-negative bacteremia.
Waked et al. 2023 [18]	2013-2020	Gram-positive	Retrospective cohort	153	111	There was no association between clinical failure and PO step-down treatment for uncomplicated streptococcal BSIs. The two groups had similar rates of 90-day mortality, hospital readmission, recurrence, and AAE.
Kang et al. 2022 [19]	2015-2017	Gram-positive	Retrospective cohort	146	98	​​In uncomplicated streptococcal BSI, patients treated with step-down PO antibiotic therapy had significantly shorter length of stay compared with continued IV therapy without compromise of clinical outcomes.
Pradubkham et al. 2022 [20]	2015-2020	Gram-negative	Retrospective cohort	410	545	IV to PO transition may be a practical approach in gram-negative BSI. Patients with Gram-negative bacteremia who have HIV infection with CD4 <200 cells/mm^3^, multidrug-resistant infections, and respiratory tract sources of infection may not be ideal candidates for this approach.
Thurber et al. 2019 [21]	2008-2016	Gram-negative	Retrospective cohort	82	264	There is no significant difference in the rate of clinical failure between patients treated exclusively with IV therapy and a PO transition strategy for Gram-negative BSI secondary to urinary tract infection.
Omrani et al. 2024 [22]	2019-2022	Gram-negative	Randomized controlled trial	85	89	In patients with *Enterobacterales* bacteremia, PO switch after initial IV antimicrobial therapy, clinical stability, and source control is non-inferior to continuing IV therapy.
Gandhi et al. 2023 [23]	2017-2019	Gram-positive	Retrospective cohort	214	94	Switch to PO step-down therapy was found to be non-inferior to IV therapy for non-staphylococcal gram-positive BSIs and may be a reasonable alternative.
Gillins et al. 2023 [24]	2018-2021	Gram-positive	Retrospective cohort	33	83	The study did not observe any cases of recurrent BSI but did observe recurrent SSTIs. Among patients with uncomplicated group A streptococcal bacteremia of an SSTI source, similar readmission and mortality rates were observed between the definitive IV and PO groups.

Results of the Meta-Analysis

**Figure 2:**
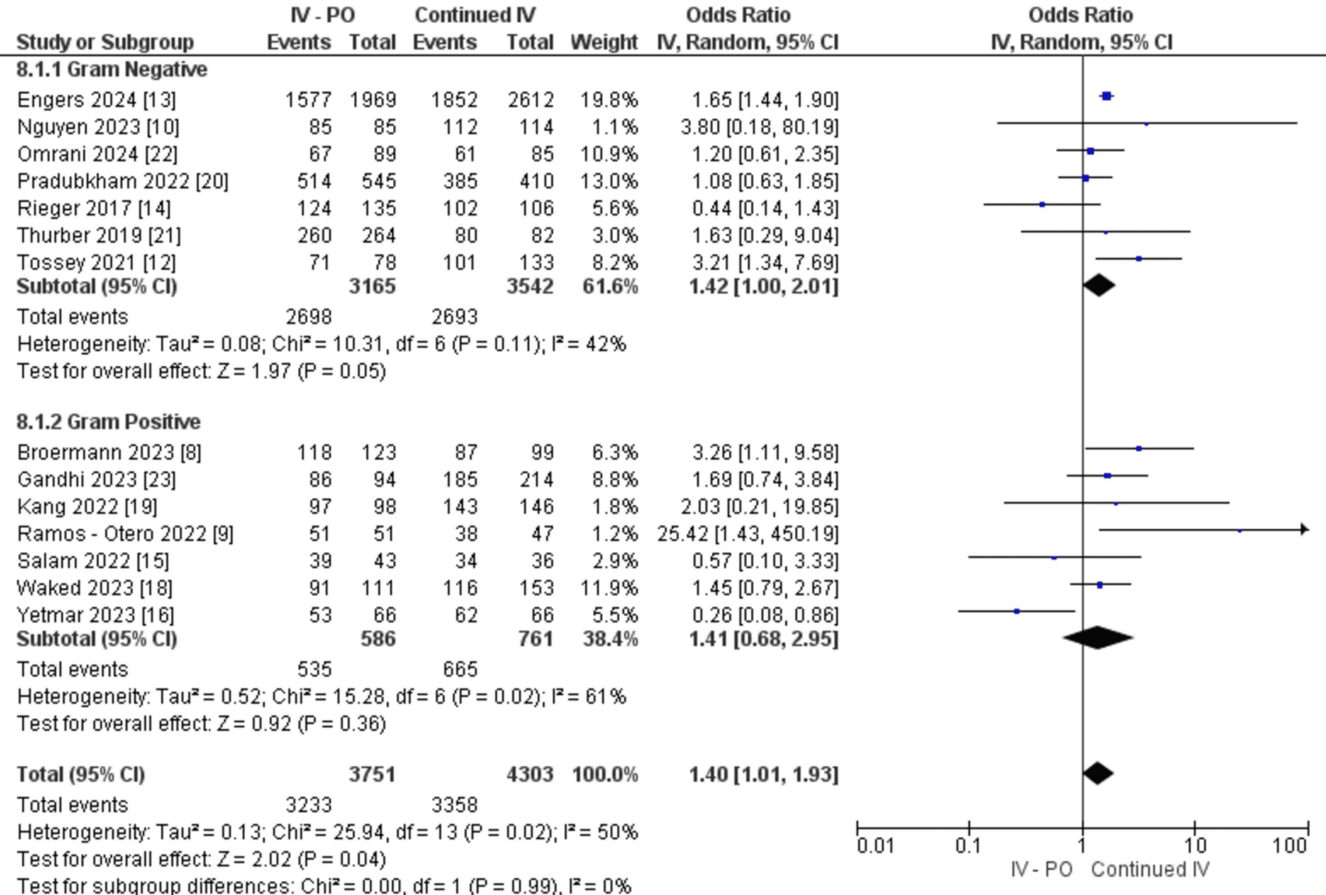
Treatment success between IV-PO and continued IV groups IV: intravenous; PO: oral

Patients who switched from IV to PO had an overall higher treatment success rate (OR: 1.40, 95% CI 1.01, 1.93, P=0.04, I2=50%). In patients with gram-negative BSI, a significantly higher treatment success was noted in patients with oral switch (OR: 1.42, 95% CI 1.00, 2.01, P=0.05). This however was not observed in patients with gram-positive BSIs (OR: 1.41, 95% CI 0.68, 2.95, P=0.36) (Figure 2).

Overall, patients who switched from IV to PO had a significantly lower risk for all-cause mortality (OR: 0.35, 95% CI 0.17, 0.69, P=0.003, I2=77%). The oral switch cohort showed a significantly lowered all-cause mortality rate in gram-negative BSIs (OR: 0.22, 95% CI 0.07, 0.67, P=0.008) but not in gram-positive BSIs (OR: 0.60, 95% CI 0.33, 1.09, P=0.09) (Figure 2). There are no subgroup differences (P=0.12).

**Figure 3:**
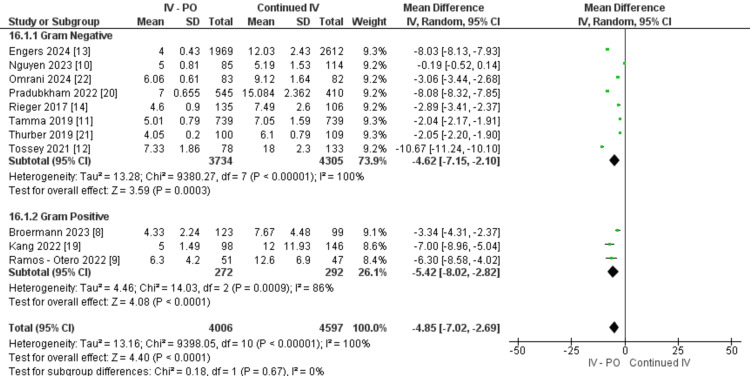
Length of hospital stay between IV-PO and continued IV groups IV: intravenous; PO: oral

The length of in-hospital stay was significantly shorter in the overall oral switch cohort (OR: -4.85, 95% CI -7.02,-2.69, P<0.0001, I2=100%). Diving into the subgroup data, both gram-negative studies (OR: -4.62, 95% CI -7.15, -2.10, P=0.0003) and gram-positive studies (OR: -5.42, 95% CI -8.02, -2.82, P<0.0000) showed significant shorter hospital stay length compared to the patients who continued IV. However, both gram-negative (P<0.00001, I2=100%) and gram-positive (P=0.0009, I2=86%) data have significant heterogeneity, but there are no subgroup differences (P=0.67) (Figure 3).

**Figure 4:**
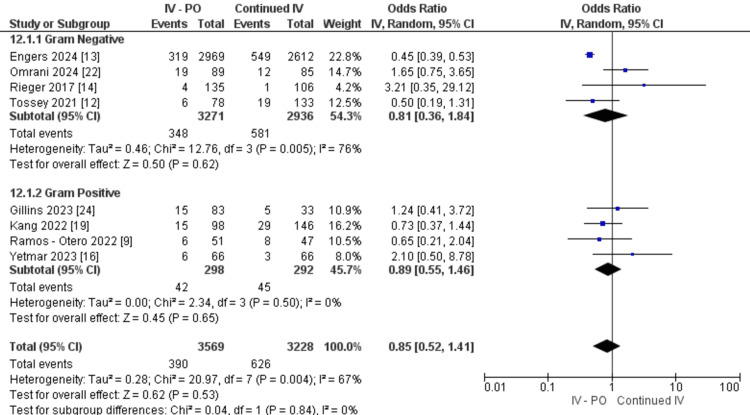
Hospital readmission rate between IV-PO and continued IV groups IV: intravenous; PO: oral

Nonetheless, compared to patients who continued IV treatment, patients who had oral switch did not show a difference in hospital readmission (OR: 0.85, 95% CI 0.52, 1.41, P=0.53, I2=67%). In both subgroups, switching from IV to PO showed no significant effect on gram-negative studies (OR: 0.81, 95% CI 0.36, 1.84, P=0.62) and gram-positive studies (OR: 0.89, 95% CI 0.55, 1.46, P=0.65) (Figure 4).

Discussion

Overview of Findings

This systematic review and meta-analysis aimed to assess the clinical outcomes of transitioning from IV to PO antibiotic therapy within the first week of treatment for BSIs compared to continued IV therapy. Overall findings of this meta-analysis revealed that patients who switched from IV to PO had a higher treatment success rate, lower all-cause mortality rate, and shorter hospital stays compared to those who continued IV treatment. However, there was no significant difference in hospital readmission rates between the two groups.

Hospital Stay

A key finding of our meta-analysis was a significantly shorter hospital stay in patients who transitioned from IV to PO antibiotics compared to those who continued IV treatment. Broermann et al. identified risk factors for prolonged hospital stay using a linear regression model, highlighting that switching to PO antibiotics independently correlated with a shorter hospital length of stay [8]. This switch signifies potential cost savings in BSI treatment and facilitates a quicker return to daily activities for patients [9].

While most studies acknowledged the cost-reducing impact of PO antibiotics, only two studies directly compared costs between IV and IV-to-PO transition groups. Nguyen et al. reported a substantial cost difference (P<0.00001) in antibiotic therapy, with median costs of 67.73 (26.03-118.05) for continued IV and 23.90 (15.68-34.47) for IV-to-PO transition groups [10]. Reduced drug preparation and administration fees, along with lower costs associated with central line placement and maintenance, contribute to these savings [11]. Regarding treatment success, our study found that patients switching from IV to PO antibiotics had a higher treatment success rate compared to those continuing with IV therapy, with an overall odds ratio of 1.40 favoring the IV-to-PO transition group. Some studies suggested that treatment failure risks in the IV group may not solely stem from the antibiotic regimen. For example, Tossey et al. identified *P. aeruginosa* bacteremia and higher Pitt bacteremia scores as significant risk factors after adjusting for confounders [12]. Subgroup analyses revealed a notable success rate in gram-negative infection studies, although discrepancies emerged. Engers et al. noted several host factors influencing treatment outcomes, reporting higher rates of immunosuppression in the IV group (31.9%) compared to the IV-to-PO transition group (24.6%) [13]. In contrast, Rieger et al. reported a superior success rate in the IV group for gram-negative infections, highlighting variation in findings across different pathogen groups [14]. In our meta-analysis, no statistically significant difference in treatment success or mortality was observed between IV-PO transition and continued IV groups for gram-positive infections, despite significant data heterogeneity. Although not statistically significant, most studies favored the success rate of the IV-to-PO approach. Salam et al. [15] and Yetmar et al. [16] were among the studies reporting higher success rates in the IV group compared to the IV-to-PO transition group.

Mortality

Included studies indicate a marginal difference between both approaches in terms of mortality. In a cohort study by Tingsgard et al., focusing on uncomplicated gram-negative septicemia, the 90-day all-cause mortality risk was 9.1% for the group undergoing step-down therapy, contrasting with 11.7% in the group subjected to continued IV treatment [17]. We hypothesize that mortality risk differs across diverse patient populations and infection types accounting for comorbidities and immune status of the included patients. This was not consistently reported across the included studies; thus, future studies are important to evaluate the course in this vulnerable population.

Another retrospective study by Tamma et al., focusing on *Enterobacteriaceae*, found that transitioning to oral therapy instead of continuing IV treatment did not show any disparity in 30-day all-cause mortality or recurrence of bacteremia concerning clinical outcomes [11]. These findings underscore the need for careful consideration of patient-specific factors and infection characteristics when determining the optimal antibiotic treatment strategy.

Readmission

In the management of BSIs, the choice of antibiotic administration method significantly impacts readmission rates. Readmissions post-bacteremia treatment can result from factors like inadequate initial therapy, antibiotic resistance, complications, immunocompromise, healthcare-associated infections, suboptimal follow-up care, or underlying medical conditions [23]. Comparing 30- to 90-day readmission rates between different administration routes helps clinicians make informed decisions for their patients. Preventing hospital readmissions following initial treatment for BSIs is crucial for both patient outcomes and healthcare system economics [24].

A study by Sessa et al. found that transitioning to oral β-lactams as part of step-down therapy increased occurrences of 30-day all-cause and infection-related readmissions in patients with *E. coli* bacteremia [25]. Furthermore, Waked et al. demonstrated comparable 90-day readmission rates between IV-only and oral step-down therapy groups in uncomplicated bacteremia [18]. This finding is further supported by a retrospective cohort study conducted by Kang et al., which indicated minimal to no difference in 30-day hospital readmission rates for uncomplicated streptococcal BSIs [19]. This finding can be logical to some extent if baseline variables are comparable in those patients. These findings underscore the importance of tailored antibiotic strategies based on the severity and profile of the BSIs to mitigate readmission risks, thereby enhancing patient outcomes and alleviating healthcare burdens.

*Strengths and L* *imitations*

Our study has several limitations worth noting. It primarily included observational studies with variable baseline patient profiles and varying criteria for transitioning from IV to PO antibiotics within the first seven days of treatment. This variability introduces biases and limits the generalizability of our findings across different patient cohorts and healthcare settings.

Additionally, differences in clinical protocols and local practices among included studies may have influenced treatment outcomes such as treatment success rates, mortality, and hospital readmission rates. The presence of selection bias, due to varied patient characteristics and severity of illness not consistently adjusted for, further complicates the interpretation of results.

Despite these limitations, our study rigorously followed systematic review guidelines and provided valuable insights into IV-to-PO antibiotic transition strategies for BSIs. Future research should address these limitations by conducting well-designed randomized controlled trials with standardized protocols to clarify optimal treatment strategies.

## Conclusions

Our meta-analysis suggests potential benefits in switching from IV to PO antibiotics early in BSI treatment where appropriate. This can contribute to high treatment success rates and shorter hospital stays. However, due to the predominance of observational studies with diverse populations and varying switch criteria, these findings should be cautiously interpreted. Future randomized controlled trials with standardized protocols are needed to validate these results and refine treatment guidelines for BSIs. Despite limitations, early oral switch shows promise in improving patient outcomes and optimizing healthcare resources.
